# Substrate mediated properties of gold monolayers on SiC†

**DOI:** 10.1039/d2ra06548g

**Published:** 2023-01-04

**Authors:** Ivan Shtepliuk, Rositsa Yakimova

**Affiliations:** a Semiconductor Materials Division, Department of Physics, Chemistry and Biology-IFM, Linköping University S-58183 Linköping Sweden ivan.shtepliuk@liu.se

## Abstract

In light of their unique physicochemical properties two-dimensional metals are of interest in the development of next-generation sustainable sensing and catalytic applications. Here we showcase results of the investigation of the substrate effect on the formation and the catalytic activity of representative 2D gold layers supported by non-graphenized and graphenized SiC substrates. By performing comprehensive density functional theory (DFT) calculations, we revealed the epitaxial alignment of gold monolayer with the underlying SiC substrate, regardless of the presence of zero-layer graphene or epitaxial graphene. This is explained by a strong binding energy (∼4.7 eV) of 2D Au/SiC and a pronounced charge transfer at the interface, which create preconditions for the penetration of the related electric attraction through graphene layers. We then link the changes in catalytic activity of substrate-supported 2D Au layer in hydrogen evolution reaction to the formation of a charge accumulation region above graphenized layers. Gold intercalation beneath zero-layer graphene followed by its transformation to quasi-free-standing epitaxial graphene is found to be an effective approach to tune the interfacial charge transfer and catalytic activity of 2D Au. The sensing potential of substrate-supported 2D Au was also tested through exploring the adsorption behaviour of NH_3_, NO_2_ and NO gas molecules. The present results can be helpful for the experimental design of substrate-supported 2D Au layers with targeted catalytic activity and sensing performance.

## Abstract 2

In light of their unique physicochemical properties two-dimensional metals are of interest in the development of next-generation sustainable sensing and catalytic applications.

## Graphical abstract

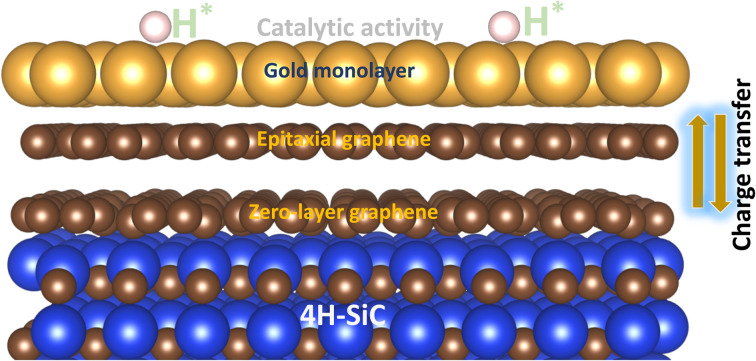

## 1. Introduction

Two-dimensional metals (2DMs) are highly promising materials for the development of novel type room temperature gas sensors with desired sensitivity and selectivity.^1–8^ Their enhanced potential for sensing is mainly governed by the unique physicochemical properties, namely the abundance of coordinatively unsaturated metal sites, which are available for adsorption of multiple gas molecules. Furthermore, the electrical conductivity of single-atom-thin metals is sensitive even to parts-per-billion concentrations of gas molecules,^4^ which makes them excellent candidates for charge transducer layers in chemo-resistive sensors. Apart from this, there are numerous reports on using ultrathin 2D nanosheets of noble metals for electrocatalysis.^9–19^ In addition to the presence of coordinatively unsaturated atoms, a transition from bulk metal to 2D metals can also cause a significant increase of the active surface area, suggesting an evident advantage over 3D bulk catalysts in terms of a surface-normalized catalytic activity and a more rational utilization of noble metals. These features highlight the importance of a thorough study of 2DM formation and the need for a deep understanding of fundamental material–property relationships of 2DMs especially in the context of realistic sensing and catalytic applications.

Large efforts are being currently invested in developing reproducible and reliable technologies for synthesis of 2D metals.^20–25^ The earliest study was related to the synthesis of 2D metals inside nanopores of graphene^20,21,25^ while more recent approaches include the stabilization of 2D metal layers (Ga, Ag, Au, Sn) under graphenized SiC surfaces *via* the intercalation process.^26–31^ In contrast, it was recently demonstrated that 2D Pt can be directly grown on top of carbon rich reconstructed SiC surface (also known as zero-graphene layer, ZLG),^4^ which is envisioned as an encouraging substrate to accommodate 2D metals. This interest is driven by the unique corrugated topology of the ZLG that has mixed sp^2^–sp^3^ chemical bonding to the SiC substrate and can provide a suitable adsorption energy of metal adatoms to promote 2D growth regime.^32^ It has been shown that directly deposited 2D Pt can be utilized for chemical sensing of benzene and formaldehyde in the part-per-billion range.^4^

Despite the progress made in addressing issues related to 2D metals formation on graphenized SiC and related applications, certain fundamentally important aspects of the nature of the substrate effect on the growth and catalytic activity of 2D metals still need to be better understood. In particular, the question whether 2DMs catalytic activity will be affected by decoupling of ZLG from SiC substrate has not been answered. Furthermore, since experimentally synthesized ZLG/SiC samples may contain both ZLG-free bare SiC and overgrown epitaxial graphene (EG) regions, different types of interfaces including Au/SiC and Au/EG/SiC should be considered, especially to make a distinction between inequivalent 2DM-based catalyst active phases.

Here we report theoretical results on the effect of the degree of graphenization of SiC on the formation of gold monolayer selected as a model two-dimensional metal. We chose the hydrogen evolution reaction (HER) as a model reaction for exploring the substrate mediated catalytic activity of 2D gold layers. The substrate effect on the HER performance is investigated with the aim to ascertain the extent of sensitivity of the free energy of hydrogen adsorption to 2D gold properties driven by metal–substrate interaction. The results presented in the paper will be of importance both for designing novel-type materials based on the beneficial combination of atomically thin metal, 2D carbon phases and robust SiC support and for future experiments in 2DMs-based sensorics and catalysis. From the experimental point of view, we ought to emphasize that the use of semi-insulating (SI) SiC substrates would facilitate the analysis of the HER performance because the electrochemical signal will be mostly generated from the surface layers of gold-decorated structures, which are in contrast highly conductive as in the case of EG/SiC. This will allow to link the changes in the interfacial chemistry induced by both graphenization and 2D gold formation to changes of hydrogen evolution reaction at the gold-decorated surface. While the conductivity of ZLG/SiC can be favorably modified by Au intercalation. We also investigate the adsorption behavior of NH_3_, NO_2_ and NO gas molecules to highlight the sensing potential of substrate-supported 2D Au.

## 2. Methods

All density functional theory (DFT) calculations have been performed using the SIESTA code.^33^ For all calculations, vdW-BH functional combined with double-ζ polarized (DZP) basis set is employed.^34^ Considering our previous experience,^32,35–37^ DZP basis set enables attaining satisfactory results for the EG/ZLG/SiC system, including the interaction between EG and the noble metals. The energy shift and force tolerance are set to be 200 meV and 0.02 eV Å^−1^, respectively. *k*-grid of 9 × 9 × 1 Monkhorst–Pack is used to sample Brillouin zone for relaxation-type DFT calculations. While for calculations of electronic and charge transfer properties, a 24 × 24 × 1 *k*-point grid is used. We apply ATOM code^38^ to generate norm-conserving Troullier–Martins pseudopotentials for C, Si, Au and H atoms. To mimic the EG, we initially construct 2 × 2 hexagonal graphene lattice on 
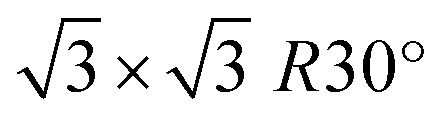
 surface reconstructed SiC (0001) that is well-recognized model of epitaxial graphene on 4H-SiC (0001).^39–41^ In turn, ZLG/SiC is modelled by removing the topmost graphene layer from the structure. To form the interface between the cubic phase of Au and hexagonal packed structure of ZLG/SiC and EG/SiC, we first transform the hexagonal 
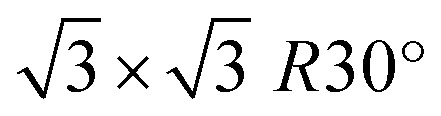
-model into a model with an orthorhombic unit cell (Fig. S1 and ESI†), which however retains the hexagonal packaging and then put the gold (111) layer onto the ZLG or EG surface. We found that the (1 × 3 × 1) gold (111) supercell matches well with the rectangular (2 × 2 × 1) supercell of EG or ZLG (Fig. S2 and ESI†). Eventually, the unit cell was duplicated twice in the in-plane directions so that the main model of the Au/ZLG/SiC and Au/EG/SiC systems encompasses a single Au monolayer containing 24 Au atoms, which is placed onto a slab surface. However, an initial smaller model was also tested. However, this model that contains nine Si–C bilayers was only utilized to investigate the adhesion of the 2D Au to the substrate and the interfacial charge transfer. While SiC (0001) substrate comprising two Si–C bilayers (Fig. S3 and ESI†) was used to scrutinize the catalytic activity and sensing potential of substrate-supported 2D Au. This enables to reduce the computational cost while remaining in compliance with the results for the thicker slab model. To simulate the Au-intercalated ZLG electrode, we perform additional calculations *via* placing eight Au atoms in the region between Au/ZLG and SiC surface. It should be mentioned that, from the thermodynamic point of view, the gold intercalation beneath ZLG is a feasible process. Recently, it was experimentally shown the principal possibility of annealing-induced gold intercalation beneath ZLG followed by the formation of quasi-free-standing monolayer graphene.^27,29^ This was achieved through thermal annealing (up to 850 °C) of the vapor-deposited gold layer on ZLG/SiC. In the present work, we consider a minimum number of intercalated gold atoms, which is enough for a complete ZLG decoupling from SiC surface and its subsequent conversion to the quasi-free standing epitaxial graphene. Although, it is expected that an increase in the amount of Au intercalants will affect the electronic properties of the whole structure and hence the catalytic and sensing performance of topmost 2D Au layer.

For better understanding of the substrate effect on the catalytic activity of gold, a free-standing 2D Au layer was additionally explored (Fig. S4 and ESI†). To avoid undesired interaction between the slab and its periodic replica, a vacuum layer of 25 Å is added above the surface along the slab-normal direction.

To estimate the interaction strength of 2D Au/support system, we estimated the Basis Set Superposition Error (BSSE)-corrected binding energy using the following equations:1
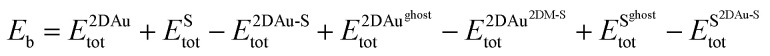
where *E*^S^_tot_ is the total energy of the isolated support (SiC, ZLG or EG), *E*^2DAu^_tot_ is the total energy of isolated 2D Au layer, and *E*^2DAu-S^_tot_ is the total energy of the 2D Au/support system. Four remaining terms of eqn (1) are utilized to estimate BSSE correction energy. 
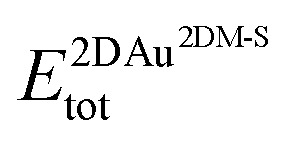
 and 
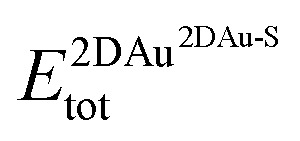
 are the total energies of the 2D Au layer and the support in the relaxed geometry of the 2D Au/support system, while 
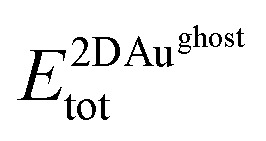
 and 
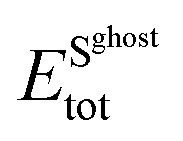
 are the total energies of the 2D Au layer and support in the relaxed geometry of the 2D Au/support system by using ghost atoms.

Rearranging eqn (1) and introducing three new terms (interaction energy component *E*^BSSE^_int_, and deformation energies *E*^2DM^_def_, *E*^S^_def_) one can assume the following:2
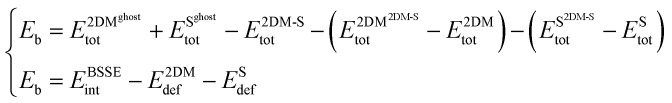


From the physical point of view, deformation energies enable estimating the energy penalties that need to be paid to accommodate 2D Au layer on a supporting substrate.

Since d-band center of the metal is an important parameter determining the HER performance,^42,43^ we calculated the d-band center by using the following expression:3
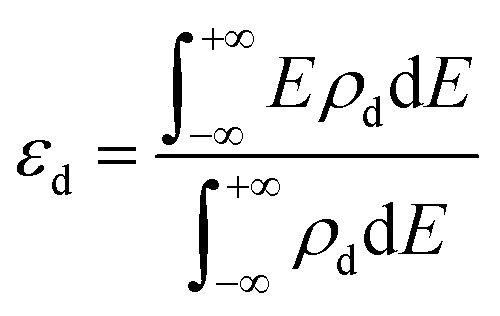
where *E* is the energy, and *ρ*_d_ is the partial density of states (PDOS) of the d-type orbitals of the gold atoms.

To examine the room-temperature (300 K) stability of 2D gold layers formed on different substrates, we performed additional molecular dynamics (MD) calculations using “anneal” thermostat implemented into the SIESTA. Temporal stability of gold layers was studied using the model containing two Si–C bilayers. A time step and a relaxation time were chosen equal to 1 and 25 fs, respectively.

Sensing potential of 2D gold layers of the slabs of two Si–C bilayers was uncovered through the investigation of the adsorption behavior of selected hazardous nitrogen-containing compound gas molecules like NH_3_, NO_2_ and NO. For this aim, Troullier–Martins pseudopotentials for O and N atoms were additionally generated before geometrical optimization procedure.

## 3. Results and discussion

### 3.1. Formation of gold monolayer on different SiC surfaces

**Fig. 1:**
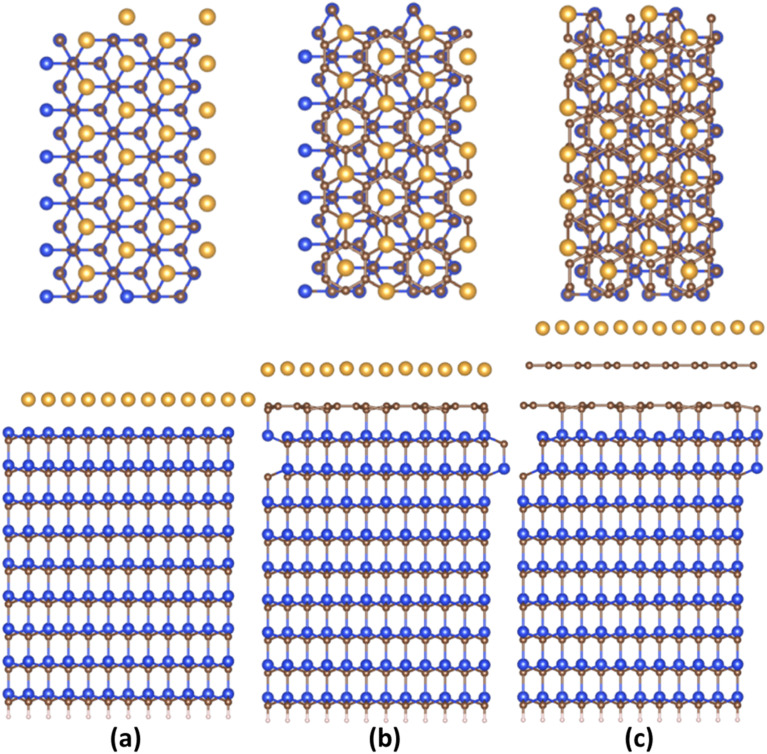
(Top and side views) Optimized structures of substrate supported 2D Au layers: (a) 2D Au/SiC, (b) 2D Au/ZLG, and (c) 2D Au/EG, respectively. Brown, yellow, blue, and whitish balls represent C, Au, Si, and H atoms, respectively.

Fig. 1 and S2 (ESI†) demonstrate the relaxed structures of 2D gold layers supported by bare SiC, ZLG and EG substrates, respectively. For the sake of comparison, two different slab models (1 × 3 × 1 and 2 × 6 × 1) were utilized. Notably, no significant effect of the slab size on the main structural parameters of gold, like average 2D Au-support distance and mean Au–Au bond length is revealed (see also Table 1). Nearly atomically flat 2D gold is formed onto the bare SiC substate, while more corrugated layers are observed onto ZLG and EG substrates. It should be also mentioned that the binding energy of 2D Au/SiC system is positive (exothermic process) and is the largest among other systems (Table 2). However, the stabilization of 2D Au layer on SiC surface is attained *via* deformation of both SiC support and 2D Au layer. The predicted values of the deformation energies are summarized in Table 2. In contrast, the energy that is required to deform 2D Au layer when interacting with ZLG and EG is significantly reduced. Much lower binding energies (which become negative) of 2D Au/ZLG and 2D Au/EG compared to 2D Au/SiC indicate that the formation of 2D Au on these supports is endothermic process and the total interaction is mainly governed by weak dispersive forces. The stability of the considered structures was then scrutinized through performing MD calculations at room temperature. The results are given in Fig. S5 and S6 (ESI†). The corrugation of the 2D Au layer (determined as a difference between maximum *z* coordinate value for Au and mean *z* coordinate value for Au) was chosen as a descriptor of the planarity of gold monolayer. It was revealed that at room temperature in all cases 2D gold remains its native planar structure. Although corrugation oscillations (not exceeding 1 Å for gold on ZLG and EG) take place, no phase transition from continuous 2D layer to 3D clusters is observed. It is interesting to note that strongly interacting bare Si-face of the SiC substrate stabilizes the gold layer, so that the atomic displacements in this case are even smaller than those in the free-standing gold layer. However, 2D-to-3D transition at higher temperatures cannot be ruled out.

**Table 1:** Parameters describing the properties of 2D Au layer

Support		Mean 2D Au-support distance, Å	Mean Au–Au bond length, Å	Corrugation of 2D Au layer, Å	Charge on 2D Au, *e*^−^	
					Hirshfeld	Voronoi
Si-face SiC	1 × 3 × 1	2.5975	3.1257	3.2832 × 10^−4^	0.2220	0.1380
	2 × 6 × 1	2.5962	3.1256	8.3861 × 10^−4^	0.8880	0.5520
ZLG	1 × 3 × 1	2.9678	3.0829	0.0577	0.0240	0.0680
	2 × 6 × 1	2.9676	3.0833	0.0595	0.0160	0.1920
EG	1 × 3 × 1	2.9518	3.0481	0.0327	0.0340	0.0600
	2 × 6 × 1	2.9453	3.0486	0.0404	0.1840	0.2880
No support	1 × 3 × 1	—	3.0069	0	0	0
	2 × 6 × 1	—	3.0069	0	0	0

**Table 2:** Parameters describing the interaction between 2D Au layer and support. Note: the absolute values of *E*^BSSE^_int_ energy component and deformation energies are listed in the table, while the binding energies are given per unit cell

Support		BSSE-corrected interaction energy, eV	Deformation energy of support, eV	Deformation energy of 2D Au, eV	Binding energy per unit cell, eV
Si-face SiC	1 × 3 × 1	5.9695	0.5717	0.7459	4.652
	2 × 6 × 1	23.9862	2.3271	2.8839	4.694
ZLG	1 × 3 × 1	−0.5024	0.0421	0.4070	−0.951
	2 × 6 × 1	−1.7162	0.1860	1.5762	−0.870
EG	1 × 3 × 1	−0.6307	0.0142	0.1881	−0.833
	2 × 6 × 1	−2.5843	0.0239	0.7256	−0.833

**Fig. 2:**
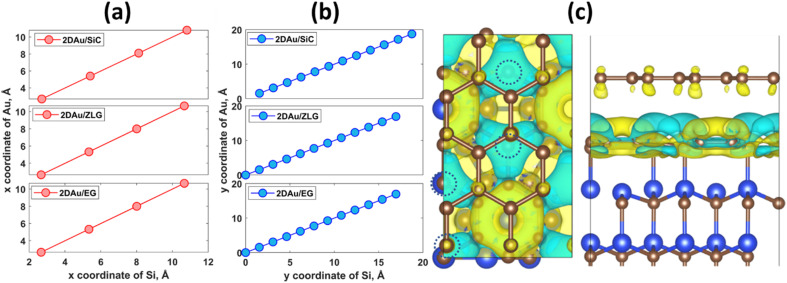
Mutual dependences of cartesian coordinates of aligned Si and Au atoms for three considered structures: (a) *x*-axis and (b) *y*-axis. (c) Charge distribution at EG/ZLG/SiC interface (top and side views). Dashed circles designate the positions of sp^3^-bonded carbon atoms of ZLG. The iso-surface level was set to 8 × 10^−4^ e Å^−3^. Yellow and cyan colors correspond to positive (charge accumulation) and negative (charge depletion) Δ*ρ*.

An important observation is that the mean Au–Au bond length tends to decrease when moving from 2D Au/SiC to 2D Au/EG. It can be stated that the 2D gold layer experience an expansive deformation when interfacing to bare SiC. While the Au–Au bond length for 2D Au/EG becomes much closer to the same parameter of support-free 2D Au. It is interesting to note that each of the gold atoms belonging to SiC-supported 2D Au layer occupies top site above surface Si atoms (so called single-coordinated on-top sites). In the case of ZLG, one part of gold atoms sits directly above sp^3^-bonded carbon atoms of ZLG, while another part of the gold atoms occupies hollow site of ZLG (centers of undistorted hexagonal rings). In turn, gold atoms on EG surface are either above bridge sites (center of the C–C bond) of the topmost graphene layer or in the close vicinity of the bridge sites. Furthermore, a more detailed analysis of the spatial arrangement of gold atoms suggests that, independently of the presence of ZLG or EG above SiC, all gold atoms on the three considered surfaces are located directly above the surface Si atoms of SiC. In this regard, mutual dependences of *x* and *y* cartesian coordinates of aligned Si and Au atoms follow a generally linear trend (Fig. 2a and b), pointing out the negligible effect of ZLG and EG on the mutual arrangement of the Si and Au atoms. Such an epitaxial alignment of gold atoms to silicon atoms of SiC substrate through zero-layer graphene and topmost graphene layer can be, to some extent, referred to the remote epitaxy concept.^44^ More particularly, it was argued that the potential field of numerous substrates cannot be fully suppressed by simply adding graphene above the substrate, which is due to the weak van der Waals potential of graphene. A direct consequence of this is a possibility of homoepitaxial or heteroepitaxial growth of different materials on graphene-covered bulk substrates. For example, homoepitaxial growth of GaAs (001) was realized on GaAs (001) substrate in the presence of the transferred graphene,^44^ while the graphene-coated *c*-Al_2_O_3_ wafer can provide pre-conditions for the heteroepitaxial growth of GaN microrods.^45^ Back to our case, to introduce the nature of the remote epitaxy applicable to our material systems we computed charge density distribution of EG/SiC substrate by subtracting the charge density of ZLG and EG layers from the total charge density (*ρ*_EG/ZLG/SiC_ − *ρ*_EG/ZLG_). Such an approach enables excluding the background charge density originating from ZLG and EG and concluding about the penetration depth of the electric attraction.^45^ As can be seen from Fig. 2c the charge accumulation occurs on undistorted hexagonal rings of ZLG, while the charge depletion regions are formed near the sp^3^ bonded carbon atoms. Notably, the net charge formed at ZLG layer is much larger than that at the topmost graphene layer, at which a slight charge accumulation is observed. This implies that the electrical attraction originating from ZLG/SiC interface may spread through graphene layer, thereby explaining the epitaxial alignment of gold atoms with respect to the Si atoms. However, these are only theoretical results and a more detailed experimental study of the long-distance ZLG (EG)-mediated interaction between gold and SiC is needed to confirm a fundamental possibility of the remote epitaxy of gold on graphenized SiC.

**Fig. 3:**
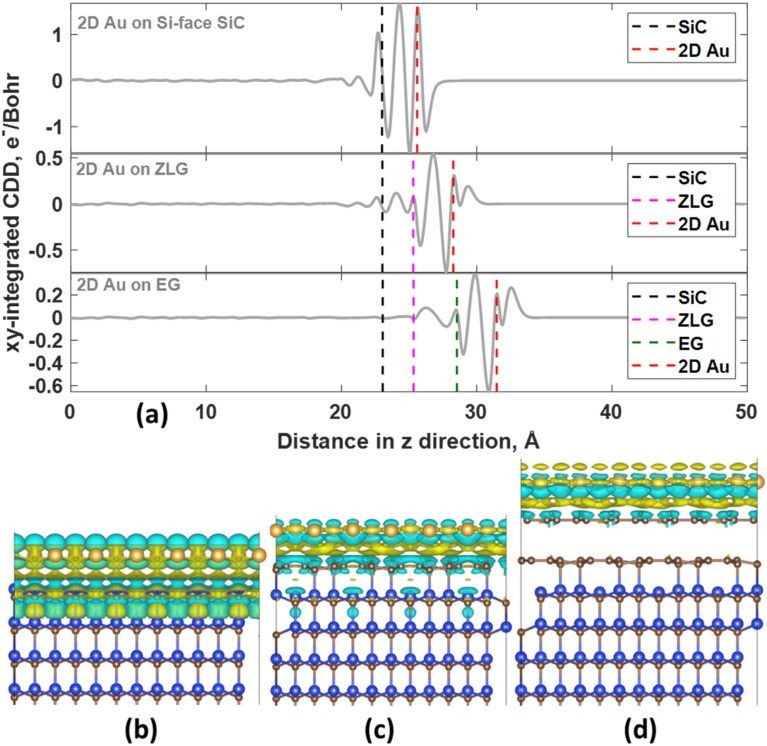
(a) The planar-averaged charge density difference for the 2D Au/SiC, 2D Au/ZLG and 2D Au/EG systems as a function of position in the *z*-direction. The dashed vertical lines correspond to the location of the topmost SiC layer, ZLG, graphene, and 2D Au layer, respectively. Positive and negative values of the CDD represent charge accumulation and charge depletion regions, respectively. 3D charge density difference (3D-CDD) characterizing Au/SiC (b), Au/ZLG/SiC (c), and Au/EG/SiC (d) interfaces, respectively. The iso-surface level was set to 5 × 10^−4^ e Å^−3^. CDD was calculated as follows: Δ*ρ* = *ρ*_Au/support_ − *ρ*_Au_ − *ρ*_support_.

In all considered cases, the 2D gold layer donates its electrons to the substrate, becoming positively charged. The charge transfer magnitude follows the order 2D Au/SiC > 2D Au/EG > 2D Au/ZLG (Table 1). This trend is consistent with the increase in the mean 2D Au-support distance from 2.59 Å for SiC to 2.96 Å for ZLG. Concomitantly, the reduced charge transfer from 2D Au to ZLG compared to other substrates can be explained by the fact that ZLG, partially covalently bonded to SiC, contains some fraction of sp^3^ bonded carbon atoms (C–Si bonds) that are not conducive to the effective electron transport. Particularly, gold atoms located directly above sp^3^-hydbridized sites of ZLG are nearly charge-neutral. The results of charge population analysis are then corroborated by the analysis of charge density difference for the 2D Au/SiC, 2D Au/ZLG and 2D Au/EG systems (Fig. 3).

More specifically, the redistribution of the net charge at the 2D Au/SiC interface includes the formation of the net charge accumulation region between 2D Au layer and SiC surface and two charge depletion regions from both sides of the 2D Au layer (Fig. 3a and b). The amount of the charge transferred from 2D Au to SiC is the largest compared to that for other systems (Fig. 3a, c and d). We also noticed that in the case of 2D Au/EG system no obvious net charge redistribution at the ZLG/SiC boundary occurs. It is worth noting the presence of a positive peak directly above the position of 2D Au in planar-averaged CDD of 2D Au/ZLG and 2D Au/EG that is absent for 2D Au/SiC system (Fig. 3a). This means the formation of charge accumulation surface region in the former case and the depleted surface region in the latter case. For 2D Au/EG, this charge accumulation region is more pronounced compared to 2D Au/ZLG system. As will be shown later, such a unique charge redistribution may influence the surface catalytic activity.

### 3.2. Catalytic activity of the substrate-supported gold monolayer

**Fig. 4:**
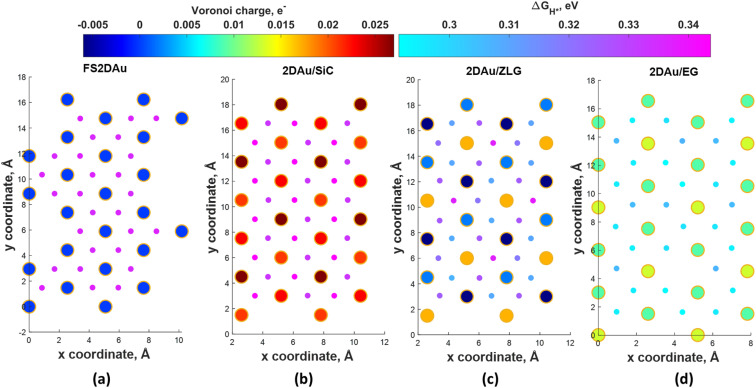
Maps of predicted free energy adsorption, Δ*G*_H*_, for the Volmer step on different model surfaces: (a) free-standing 2D Au, (b) 2D Au/SiC, (c) 2D Au/ZLG and (d) 2D Au/EG, respectively. Small circles correspond to Δ*G*_H*_ values for H adsorbates at different sites. Δ*G*_H*_ was calculated by using the following equation: Δ*G*_H*_ = Δ*E*_H*_ + 0.24 eV (ref. 46), where Δ*E*_H*_ is the adsorption energy of hydrogen, while the term 0.24 eV represents the energy changes in zero-point energy (ZPE) and entropy between the gaseous H and absorbed H. Large circles represent magnitudes of Voronoi charge on Au atoms belonging to 2D Au layer.

To ascertain the substrate effect on the catalytic activity of 2D Au layer, we investigated the Volmer reaction (that is the first step of the hydrogen evolution reaction) on 2D Au/SiC, 2D Au/ZLG and 2D Au/ZLG surfaces through estimating Gibbs free energy of hydrogen adsorption (Δ*G*_H*_). For comparison, the results for support-free 2D Au are also given. First, the barycenter of a triangular hollow site of the 2D Au layer for all considered structures is identified as the most favorable adsorption site for hydrogen. With this in mind, we then computed the Δ*G*_H*_ for all possible barycenter sites and constructed the corresponding Δ*G*_H*_ maps combined with Voronoi charge maps for the topmost 2D Au layer (Fig. 4). Since the free-standing 2D Au is charge-neutral, all adsorption positions are equivalent and Δ*G*_H*_ is approximately equal to 0.33 eV (Fig. 4a). The underlaying SiC substrate causes charge imbalance in 2D Au and the appearance of two types of adsorption sites with Δ*G*_H*_ of 0.33 eV and 0.34 eV (Fig. 4b). The slightly increased Δ*G*_H*_ for 2D Au/SiC can be explained by the formation of the charge depletion region as was shown by 3D CDD analysis (Fig. 3b). This finding gives hints about the relationship between “charge” status of the 2D Au surface and the catalytic activity. Indeed, a more pronounced charge imbalance observed for 2D Au/ZLG system followed by the formation of charge accumulation region above 2D Au layer (Fig. 3a) results in the reduction of Δ*G*_H*_ compared to that for support-free 2D Au (Fig. 4c). In this case, Δ*G*_H*_ is ranging from 0.30 eV to 0.33 eV.

**Fig. 5:**
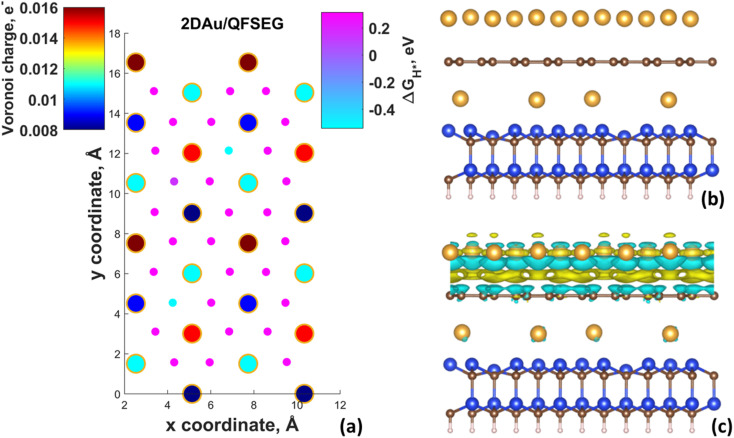
(a) Map of predicted free energy adsorption, Δ*G*_H*_, for the Volmer step on different 2D Au/QFSEG. Small circles correspond to Δ*G*_H*_ values for H adsorbates at different sites. Large circles represent magnitudes of Voronoi charge on Au atoms belonging to 2D Au layer. Optimized structure of 2D Au/QFSEG (a) and corresponding 3D-CDD (c). The iso-surface level was set to 5 × 10^−4^ e Å^−3^.

Fig. 4d demonstrates Δ*G*_H*_ maps of the 2D Au/EG system. Since CDD analysis showed the existence of charge accumulation region (even larger than in the case of 2D Au/ZLG system), an expected reduction of Δ*G*_H*_ to 0.29 eV is observed, implying an improvement of H adsorption behavior on the Au/EG surface during the HER process. The same trend was reported for Pt (111), for which HER catalytic activity was improved due to electron accumulation at Pt (111) surface.^47^ The fact that the 2D Au layer formed on graphene demonstrated better catalytic activity than 2D Au/ZLG is tempting to think that catalytic activity of 2D Au/ZLG can be tuned through decoupling of ZLG from SiC surface followed by its transformation to quasi-free standing epitaxial graphene (QFSEG) layer. In line with this we modelled 2D Au/QFSEG system through the Au intercalation process and investigated HER at its surface. Δ*G*_H*_ for all possible adsorption site is summarized in Fig. 5a.

While the optimized structure and 3D CDD of 2D Au/QFSEG system is demonstrated in Fig. 5b and c. We observed a completely different picture of hydrogen adsorption. More specifically, a substantial Δ*G*_H*_ inhomogeneity including the appearance of adsorption sites with Δ*G*_H*_ < 0 and Δ*G*_H*_ > 0 occurs. This can be related to the unique charge transfer properties at the interface mediated with Au intercalants (Fig. 5c). Indeed, the analysis of the charge density difference image shows a significant charge redistribution at 2D Au/ZLG/SiC interface due to Au intercalation, which causes an appearance of net charge accumulation above the Au layer and hence an appearance of the adsorption site with Δ*G*_H*_ = 0.14 eV. Concomitantly, the presence of two adsorption sites with Δ*G*_H*_ = −0.51 and −0.54 eV indicates that Volmer reaction at these positions is exothermic (thermodynamically favourable process).

**Fig. 6:**
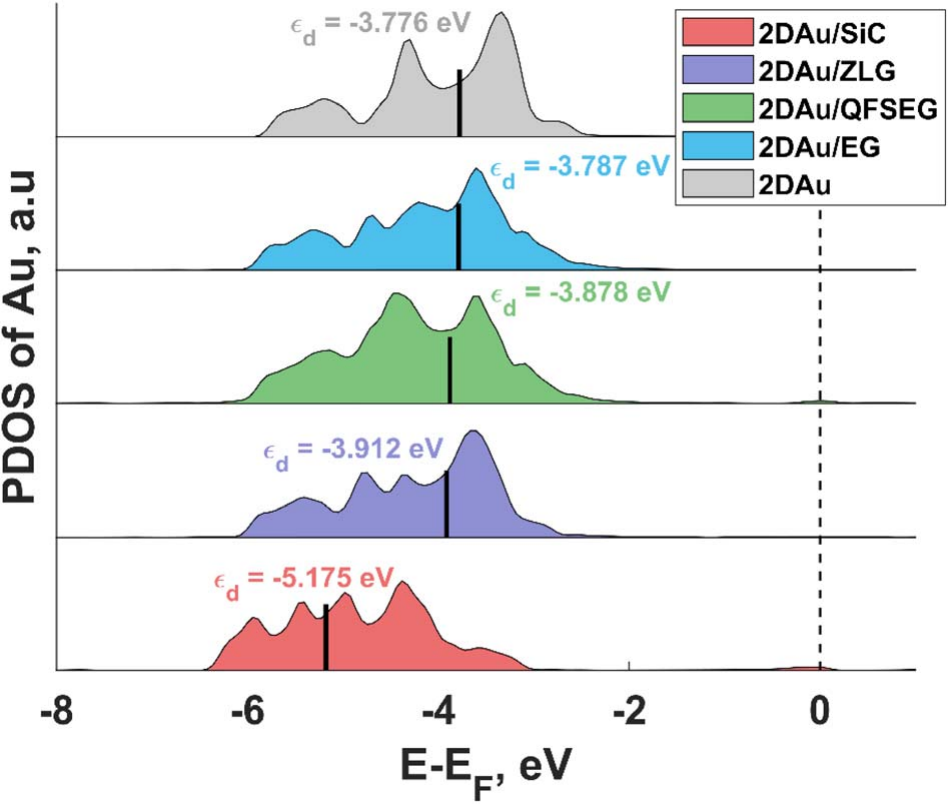
The projected d band density of states (DOS) for gold in 2D Au, 2D Au/SiC, 2D Au/ZLG, 2D Au/QFSEG and 2D Au/EG systems. Short black vertical lines designate the position of d band centre. The Fermi level is set to zero and indicated by the black dashed line.

To link the electronic properties to the catalytic activity, we also calculate partial density of states (PDOS) of 2D Au for the structures investigated herein. Since the position of the d-band center is considered as an important descriptor of HER catalytic performance, we focus on the analysis of d-orbital band of 2D Au that encompasses five projected orbitals d_*xy*_, d_*x*^2^−*y*^2^_, d_*xz*_, d_*yz*_, and d_*z*^2^_. We notice that the d-band center initially positioned at −5.175 eV (with respect to the Fermi level) for the 2D Au/SiC system exhibits an upward shift when moving from 2D Au/SiC to free-standing 2D Au (Fig. 6), implying an enhancement of the interaction between H and Au atoms. In principle, this might be beneficial for the optimization of Δ*G*_H*_ of the 2D Au. At a first glance, based on analysis of d-band centre evolution it is reasonable to assume that the less negative value of *ε*_d_ for support-free 2D Au ought to be a reason for improved Δ*G*_H*_. However, this is not necessarily the case, especially since the systems (2D Au/ZLG, 2D Au/EG and 2D Au/QFSEG) exhibiting more negative *ε*_d_ are more catalytically active towards HER. This further underlines the important role of the substrate-related interfacial charge redistribution in the adsorption of hydrogen. From Fig. 6 it is also seen that d band center can be tuned by the graphenization and the intercalation degrees control to reach even more conducive value of *ε*_d_.

Notably, among all considered cases the d band center of 2D Au on the bare SiC reaches the most negative values of −5.18 eV. The reason lies in the significant charge depletion in 2D Au layer interfacing with SiC (see Table 1 for more details) that leads to a steep decrease of the density of states near the Fermi level and an increase of the number of filled antibonding states. This increased occupancy of the antibonding states may explain the reduced catalytic activity of the SiC supported 2D Au towards hydrogen evolution reaction. Similar correlation between the interfacial charge transfer and the d-band center position was also reported for Pt-containing interfaces.^48,49^

### 3.3. Sensing performance of the substrate-supported gold monolayer

**Fig. 7:**
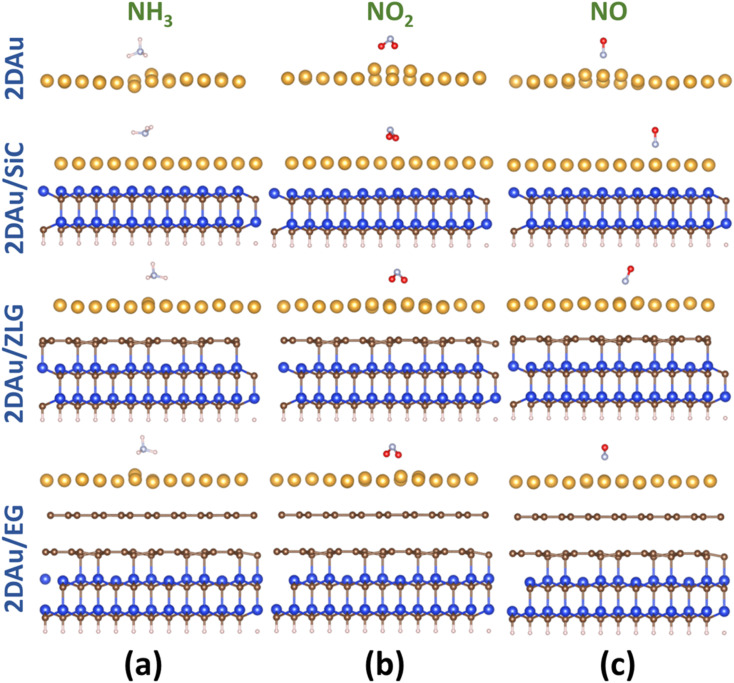
Side views of the optimized structures of 2D Au, 2D Au/SiC, 2D Au/ZLG and 2D Au/EG (from the top to the bottom) with adsorbed gas molecules: (a) NH_3_, (b) NO_2_, and (c) NO, respectively. Blue, brown, yellow, whitish, pale blue and red balls designate Si, C, Au, H, N and O atoms, respectively.

As was already mentioned in the Introduction section, due to the abundance of coordinatively unsaturated metal sites, gold monolayer is a highly promising material for the development of highly sensitive chemoresistive gas sensors. The fundamental detection principle behind 2D Au-based chemoresistive sensors lies in the chemical interaction between gas molecules and 2D metal surface which produces or consumes electrons. This strongly influences the measurable electrical properties of 2D gold layer, such as the resistance. To show its capacity as a sensor and to link the sensing performance to the substrate effect, we theoretically investigated the adsorption behavior of selected gas molecules (NH_3_, NO_2_, NO) on the free-standing gold monolayer and substrate-supported 2D Au, respectively. The choice of these hazardous gases as model analytes is justified by the urgent necessity of their detection especially for the environmental monitoring, chemical processing, and health security.^50^Fig. 7 demonstrates the side views of the considered structures after the adsorption of the different gas molecules (top views can be additionally seen in Fig. S7 and ESI†). A quick look at the adsorption configurations makes clear a significant substrate effect on the interaction between gold monolayer and gases. Particularly, in the absence of the underlying substrate the 2D Au layer undergoes substantial deformation to accommodate NH_3_, NO_2_ and NO molecules. This deformation manifests as the distinct local atomic protrusions. In contrast, 2D Au on SiC – the system with the largest binding energy – remains nearly flat after the adsorption of molecules. As the graphenization degree is increased, the gold–substrate interaction tends to decrease and 2D gold becomes more corrugated upon the gas adsorption.

**Fig. 8:**
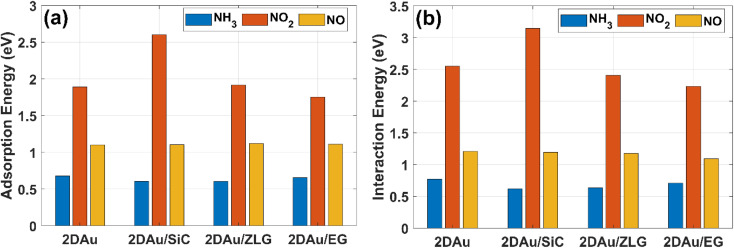
Comparative bar charts of (a) adsorption energy of gas molecules on different surfaces and (b) interaction energy between gas molecules and gold monolayer, respectively.

**Fig. 9:**
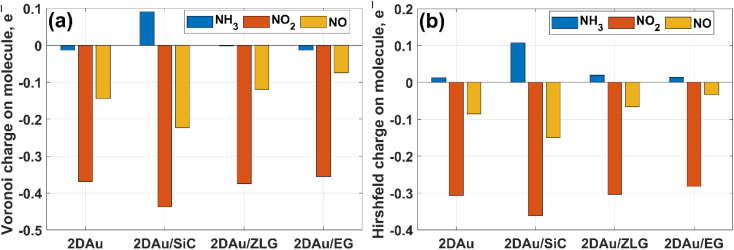
Comparative bar charts of remaining charge on adsorbed molecules calculated by (a) Voronoi and (b) Hirshfeld schemes, respectively.

We then estimate the adsorption and interaction energies for the considered systems with NH_3_, NO_2_ and NO adsorbates by using previously reported approach.^51^ Both energies demonstrate a similar trend (Fig. 8), but the interaction energies in all cases are however larger than the corresponding adsorption energies. This is because the interaction energy includes an additional energy component that is related to the deformation energy.^51^ In fact, it reflects the energy penalty that is required to be paid to host the gas adsorbates onto the substrate surface. Gold monolayer in all cases exhibits an enhanced adsorption capability to NO_2_. Therefore, it is reasonable to assume that 2D Au on SiC surfaces can be selective for NO_2_. It is interesting to note that the adsorption energy of the NO_2_ molecule depends on the graphenization degree: when moving from bare SiC to epitaxial graphene/SiC *E*_ads_ decreases and becomes comparable to that for free-standing 2D Au. This hints that the largest positive charge localized at the bare SiC (see Table 1) may favour overlapping of the metal states and the O 2p states,^52^ thereby leading to the enhanced interaction between 2D Au/SiC system and NO_2_ molecule. These results are further corroborated by the charge population analysis (Fig. 9). NO_2_ adsorbed onto 2D Au is negatively charged, suggesting an effective abstraction of electrons from the gold monolayer. The most pronounced charge transfer occurs at the NO_2_/2D Au/SiC interface. Such an enhanced charge transfer is a good prerequisite of designing fast-response NO_2_ sensors. Much weaker substrate effect is observed in the case of NH_3_ and NO adsorption. Apparently, the adsorption energy of NH_3_ in all cases is approximately 0.6 eV. This may indicate that NH_3_ molecule is physically adsorbed onto the gold monolayer. In the case of 2D Au/SiC, NH_3_ molecule donates 0.1*e*^−^ to the gold surface. While the charge transfer between NH_3_ molecule and other surfaces is negligibly small. In turn, adsorption behaviour of NO molecule resembles, to some extent, the NO_2_ case, with one exception – the adsorption (interaction) energy and charge transfer are far below those for NO_2_. Considering the fact that the adsorption energy can be correlated with the recovery time (another important descriptor of the sensor performance), one can expect the following order of desorption rate: NH_3_ > NO > NO_2_. Our finding that the graphenization degree affects both adsorption energies and the charge transfer is a good precondition for designing a gas sensor possessing a short recovery time and a fast response, and enabling a discriminative analysis.

## 4. Conclusions

By performing first principles calculation, we have revealed the substrate effect on the structural and electronic properties of a 2D Au layer deposited on SiC supports. Non-graphenized and graphenized SiC surfaces have been thoroughly investigated for their ability to accommodate gold layers. Epitaxial alignment of the gold atoms with respect to the Si atoms was revealed, irrespective of the presence of ZLG and EG. A possible explanation of this phenomenon implying the formation of long-range attraction forces acting over ZLG and EG layer was proposed. This finding may have implications in epitaxial growth of strongly lattice mismatched materials and thus facilitate utilization of such material systems in *e.g.*, sensorics and catalysis.

By comparing the substrate characteristics of SiC, ZLG and EG, including intercalation of the ZLG, we elucidated the decisive role of the interlayer charge redistribution in the adsorption of hydrogen on 2D Au. It was evidenced that the extent of graphenization significantly impacts the charge density of 2D Au and hence the HER catalytic performance as judged by Δ*G*_H*_ evolution. We revealed the correlation between charge accumulation above the Au layer and Gibbs free energy of hydrogen adsorption. This additionally highlights the prominent substrate–metal interaction strength effect on the catalytic activity of 2D Au layer. The present results gain insights into the nature of catalytic activity of the substrate-supported 2D Au and could be beneficial for the design of novel 2D electrocatalysts which in a long run may replace the expensive and less-common metals.

It was revealed that the 2D Au-based sensors are promising for detection of hazardous nitrogen-containing compound gases because of the advantageous combination of significant charge transfer and hence the expected fast response time and a selectivity.

Furthermore, the fact that thin gold layers are of importance to the field of spin transport^53^ implies that a winning combination of atomically thin gold and graphene may also boost the development of novel-type applications beyond the already proposed graphene-based spintronic devices.^54,55^

## Conflicts of interest

There are no conflicts to declare.

## Supplementary Material

RA-013-D2RA06548G-s001
