# Supplementary material for: Substrate mediated properties of gold monolayers on SiC
Source: RSC Adv. 2023 Jan 4;13(2):1125–36. doi: 10.1039/d2ra06548g (PMC9811659; doi:10.1039/d2ra06548g)
Supplement: RA-013-D2RA06548G-s001 [file RA-013-D2RA06548G-s001.pdf]

Electronic Supplementary Material (ESI) for RSC Advances.
This journal is © The Royal Society of Chemistry 2022

Electronic Supplementary Information (ESI)

for

Substrate mediated properties of gold monolayer on SiC

Ivan Shtepliuk and Rositsa Yakimova

Semiconductor Materials Division, Department of Physics, Chemistry and Biology-IFM,

Linköping University, S-58183 Linköping, Sweden

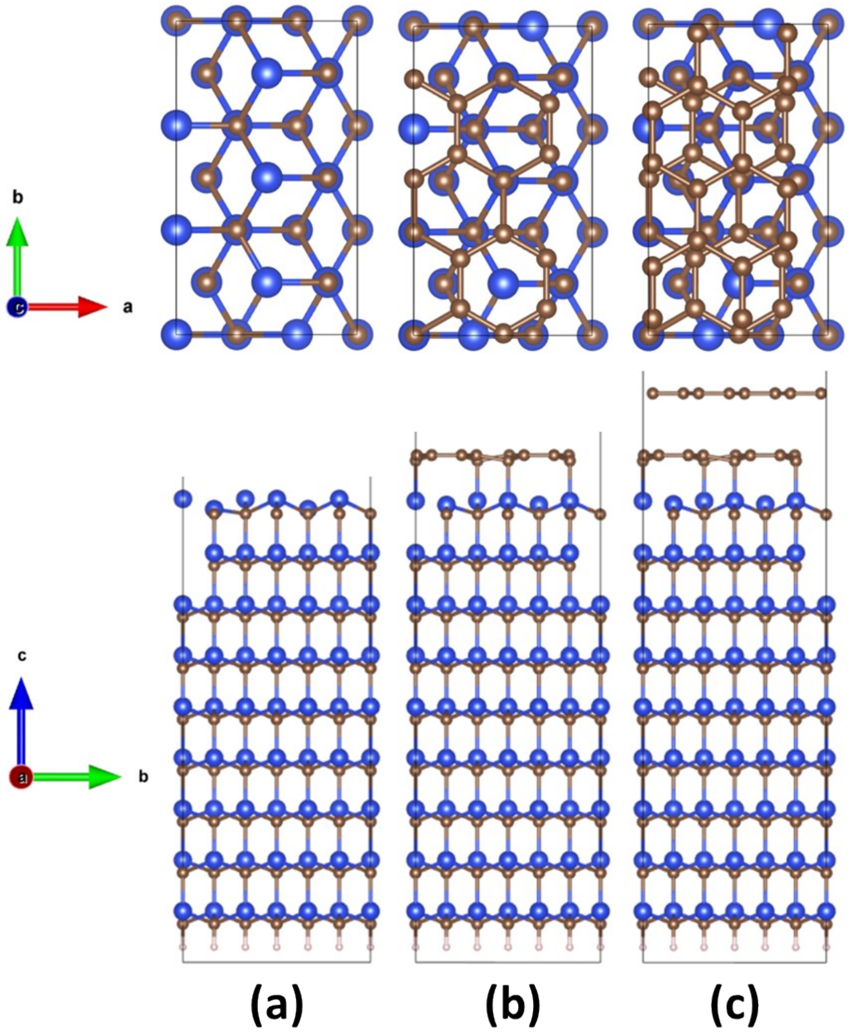

Figure S1. Top and side views of rectangular (2 × 2 × 1) supercell of SiC (a), ZLG (b) and EG (c), respectively. Brown, blue, and whitish balls represent C, Si, and H atoms, respectively.

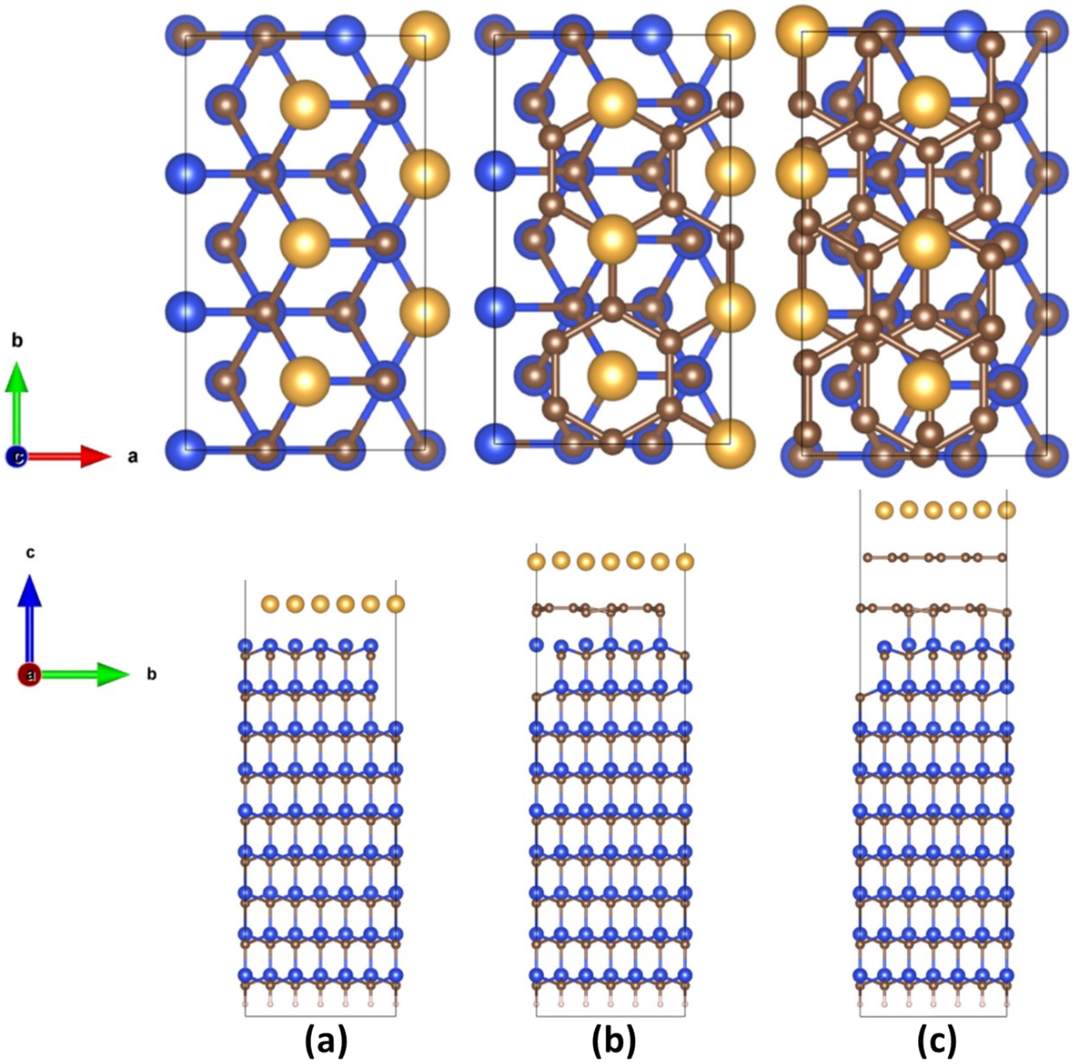

Figure S2. Construction of the gold monolayer-support interface. Top and side views of (1 × 3 × 1) gold (111) on 2 × 2 × 1 SiC (a), 2 × 2 × 1 ZLG (b) and 2 × 2 × 1 EG (c), respectively. Brown, yellow, blue, and whitish balls represent C, Au, Si, and H atoms, respectively.

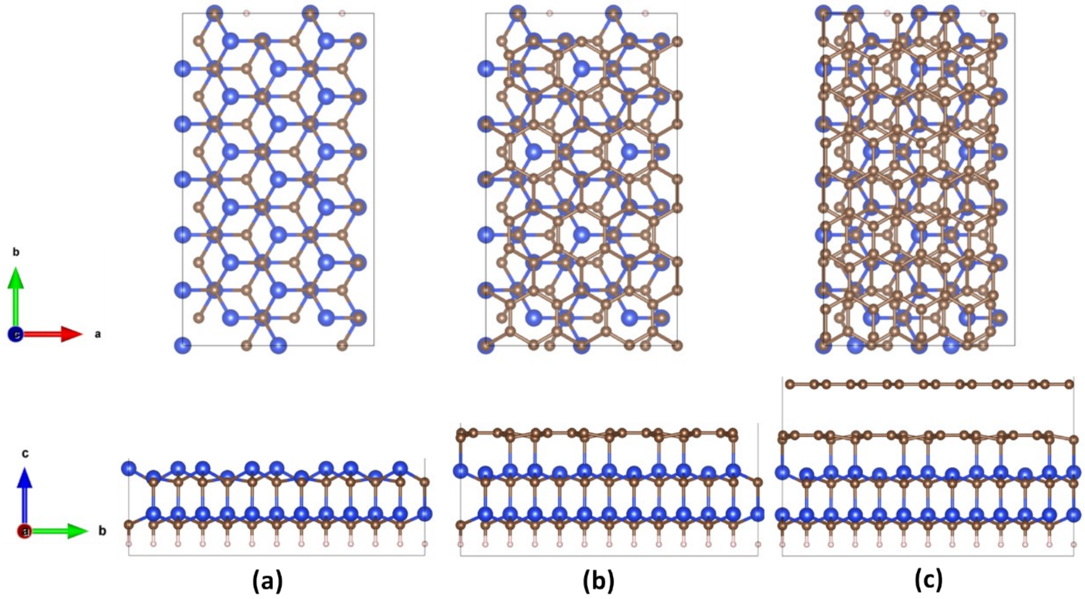

Figure S3. Top and side views of optimized structures of Substrates with two bilayers Si-C bilayers: SiC (a), ZLG (b) and EG (c), respectively. Brown, blue, and whitish balls represent C, Si, and H atoms, respectively.

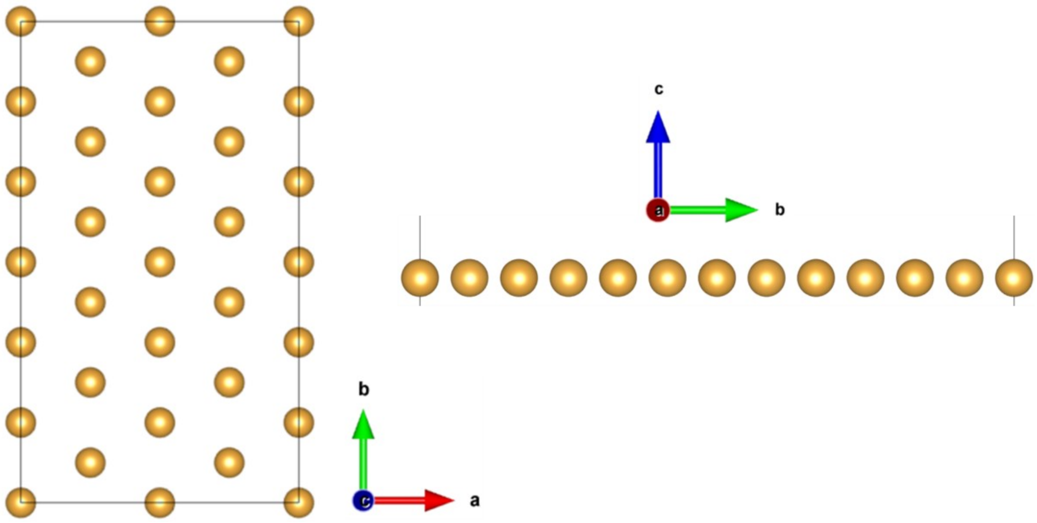

Figure S4. Top and side views of optimized structure of free-standing 2D Au layer (doubled $1 \times 3 \times 1$ supercell). Note: the real number of gold atoms is 24. While the larger number of atoms is visible in the figure due to boundary conditions.

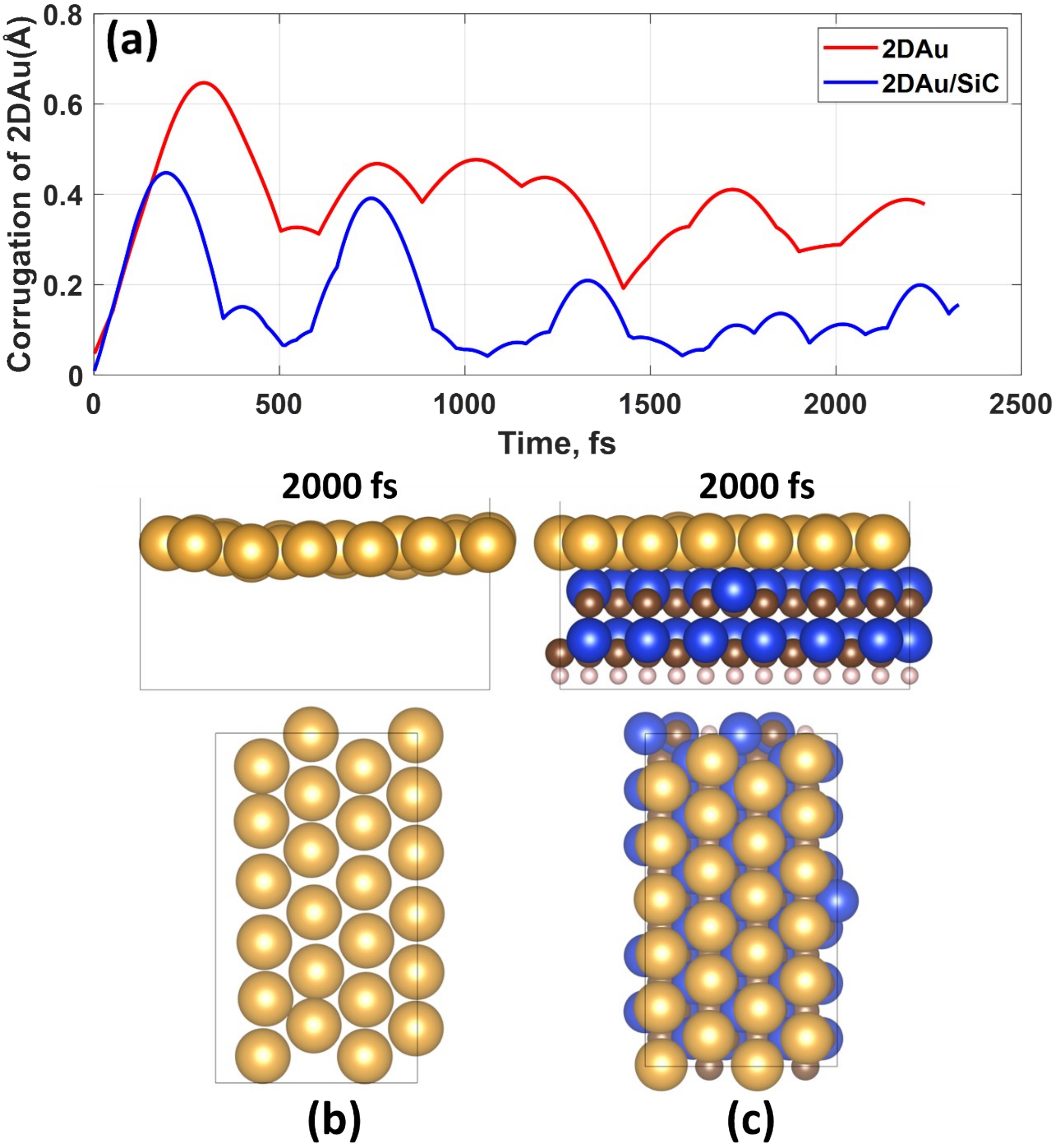

Figure S5. (a) Time evolution of the corrugation parameter of free-standing 2D Au and 2D Au on SiC, respectively. Snapshots from the molecular dynamics simulations at 300 K: (a) free-standing 2DAu and (b) 2DAu/SiC at t=2000 fs.

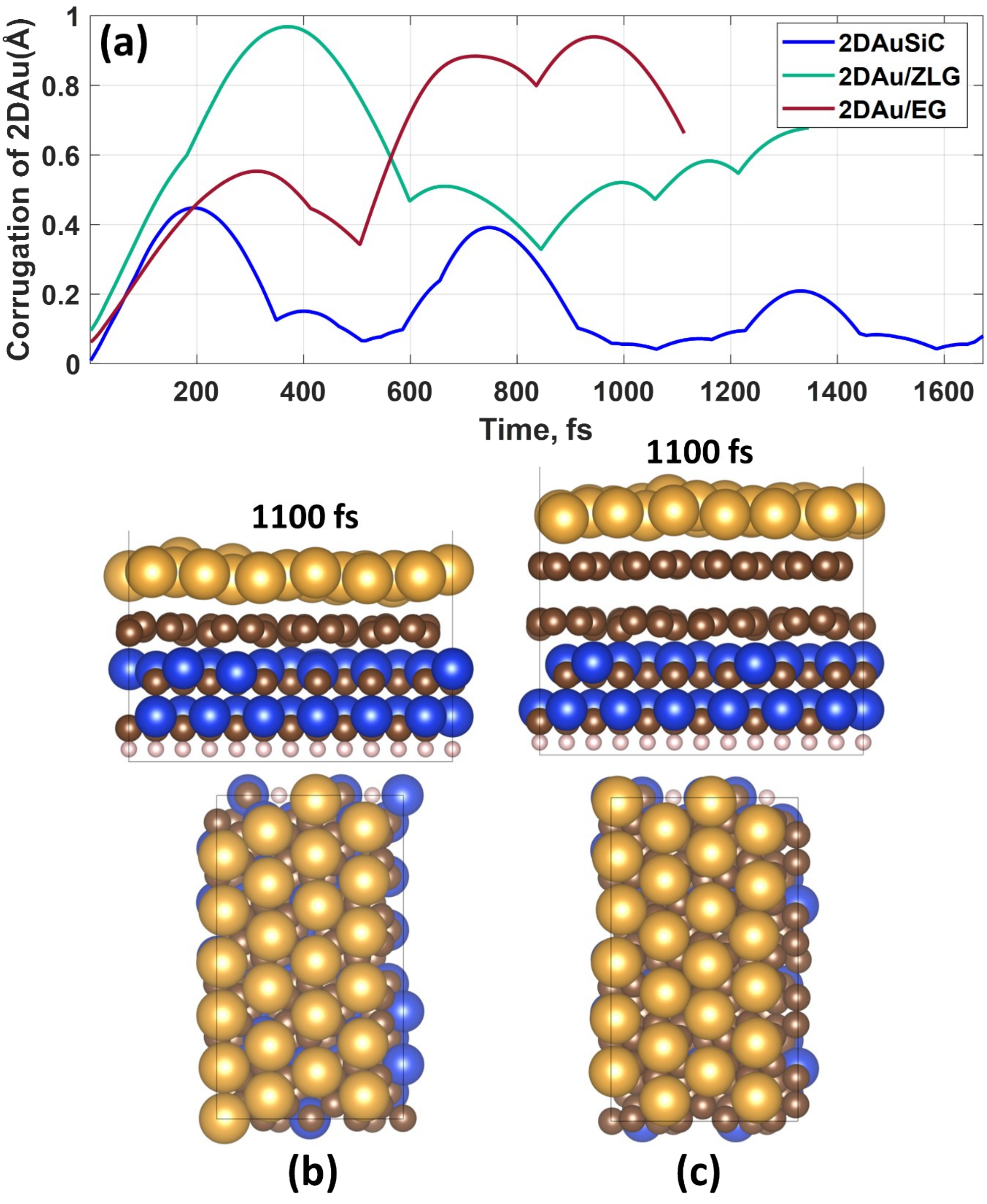

Figure S6. (a) Time evolution of the corrugation parameter of 2D Au on SiC, ZLG and EG, respectively. Snapshots from the molecular dynamics simulations at 300 K: (a) 2DAu/ZLG and (b) 2DAu/EG at t=1100 fs.

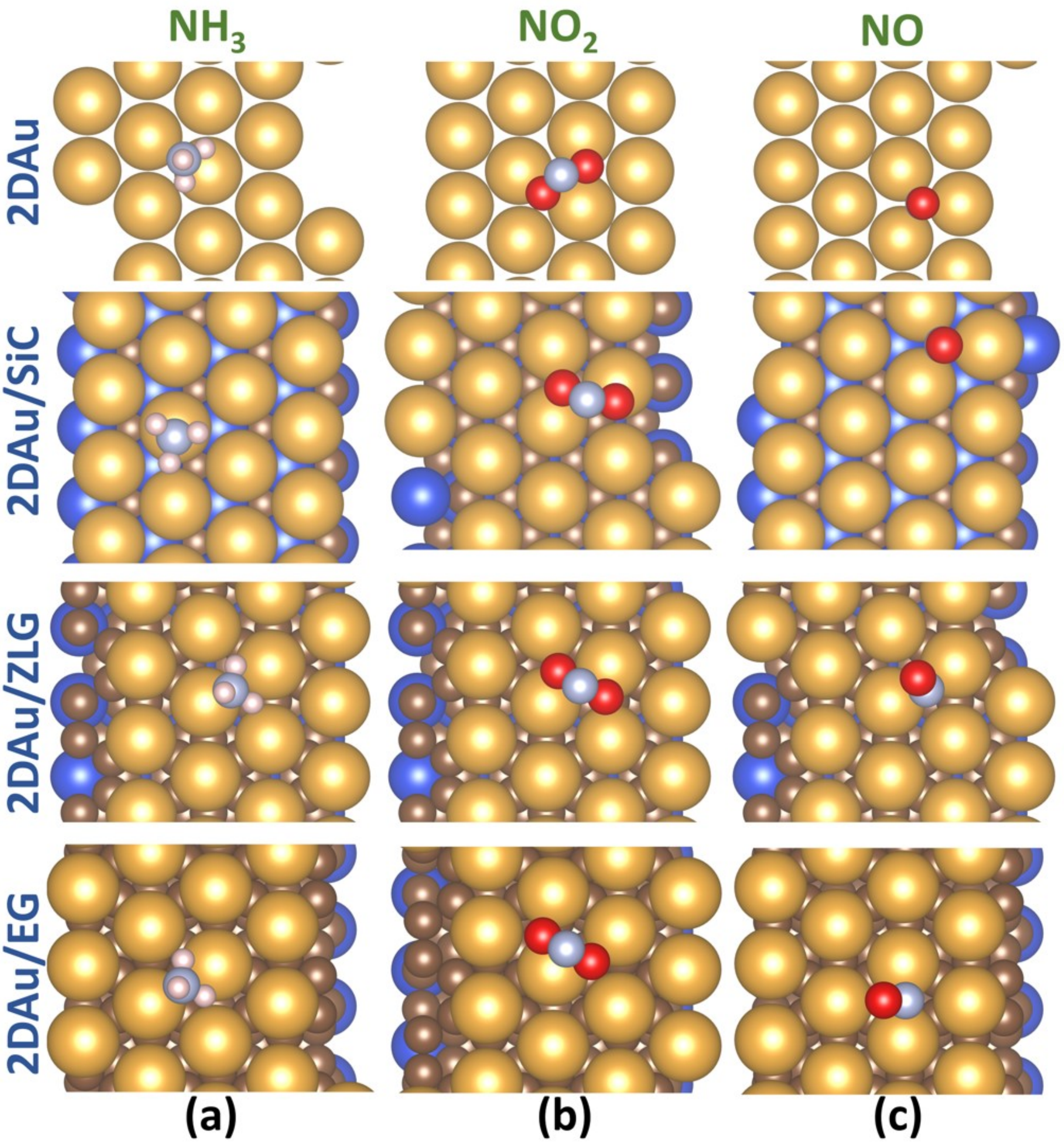

Figure S7. Top views of the optimized structures of 2DAu, 2DAu/SiC, 2DAu/ZLG and 2DAu/EG (from the top to the bottom) with adsorbed gas molecules: (a) NH_3, (b) NO_2, and (c) NO, respectively. Blue, brown, yellow, whitish, pale blue and red balls designate Si, C, Au, H, N and O atoms, respectively.
